# High resolution measurement of membrane receptor endocytosis

**DOI:** 10.14440/jbm.2018.266

**Published:** 2018-12-12

**Authors:** Zhihui Zhang, David K. Heidary, Christopher I. Richards

**Affiliations:** Department of Chemistry, University of Kentucky, 505 Rose Street, Lexington, KY 40506, USA

**Keywords:** Dendra2, fluorescence microscopy, membrane protein half-life, TIRF

## Abstract

We present a new approach to quantify the half-life of membrane proteins on the cell surface, through tagging the protein with the photoconvertible fluorescent protein, Dendra2. Upon exposure to 405 nm light, Dendra2 is photoconverted from green to red emission. Total internal reflection fluorescence microscopy (TIRF) is applied to limit visualization of fluorescence to proteins located on the plasma membrane. Conversion of Dendra2 works as a pulse chase experiment through monitoring only the population of protein that has been photoconverted. As the protein is endocytosed the red emission decreases due to the protein leaving the TIRF field of view. This method is not impacted by the insertion of new protein into the plasma membrane as newly synthesized protein only exhibits green emission. We used this approach to determine the half-life of ENaC on the plasma membrane illustrating the high temporal resolution capability of this technique compared to current methods.

## INTRODUCTION

Many different types of membrane proteins regulate several aspects of cellular function and are responsible for converting extracellular stimuli into intracellular signaling events. A wide variety of membrane proteins take part in signal transduction including ligand-gated ion channels, voltage-gated ion channels, transporters, and G-protein coupled receptors (GPCRs) [1,2]. Cell signaling initiated through these proteins is regulated by factors such as ligand-protein affinity and the number of membrane receptors on the cell surface. Thus, one way cells regulate signaling events is by controlling the level of membrane receptors on the cell surface. Ligand binding to the extracellular domain of the protein often leads to membrane receptor downregulation through endocytosis [3]. Additionally, changes in the residence time of membrane proteins on the cell surface are connected to a variety of diseases further indicating the importance of measuring these phenomena [4].

Experimental methods that can monitor the levels and duration of protein residence at the cell surface can be used to elucidate cellular function, the response to external stimuli, and to screen for therapeutics that alter these properties. Techniques to measure the half-life of plasma membrane proteins on the cell surface include surface biotinylation with western blot analysis, ligand binding, and functional studies such as electrophysiology or calcium imaging [5-8]. These techniques suffer from low temporal resolution of membrane receptor half-life and are often low throughput. Additionally, the procedure is complicated by the need to use translation blockers or intracellular protein trafficking inhibitors to eliminate the insertion of newly trafficked protein into the plasma membrane [9-11]. This also increases the error as translation blockers could affect other cellular processes that may relate to membrane protein residence time at the cell surface. This has led to varying reports of receptor half-life on the plasma membrane for some types of proteins. For example, the half-life of epithelial sodium channels (ENaC) was reported as 3.5 h following puromycin, brefeldin A (BFA) and nocodazole treatment, while the ENaC half-life was closer to 1.5 h following cycloheximide treatment [9].

Here, we introduce a total internal reflection fluorescence microscopy (TIRFM) approach in which the half-life of a membrane receptor is determined using a pulse chase method based on a photoconvertible fluorophore. Using this system, we demonstrate the measurement of changes in membrane receptor half-life with high temporal resolution for different ENaC assemblies including those that contain a mutation linked to Liddle’s syndrome [12,13].

The method utilizes a membrane protein fused with the photoconvertible fluorescent protein Dendra2, which can be irreversibly photoconverted from green to red emission upon exposure to 405 nm light [14-16]. ENaC-Dendra2 expressing cells were exposed to 405 nm light in a TIRF orientation, limiting the photoconversion to a narrow region at the plasma membrane. The red emission from the photoconverted Dendra2 labeled protein was measured with a 561 nm TIRF excitation volume to excite only the protein at the plasma membrane. The half-life of ENaC on the plasma membrane was determined by measuring the intensity of red emission over time. The new synthesized ENaC inserted to the plasma membrane during real-time imaging doesn’t disturb the measurement since it exhibits no red fluorescence. This approach enabled us to modify the temporal resolution to match the turnover rate with our highest resolution limited only by the maximum frame rate of the camera (~25Hz). This method utilizes a standard objective-based TIRF microscopy set up with a straight-forward experimental procedure.

## MATERIALS AND METHODS

### Plasmid constructs

Full length SCNN1A, SCNN1B and SCNN1G cDNA were purchased from Origene and cloned in pcDNA 4TO. The fluorescent protein Dendra2 was incorporated in to the C-terminus of each construct *via* NotI and XhoI restriction sites. The quickchange II site-directed mutagenesis kit (Agilent Technologies) was used to create the Y618A mutation in SCNN1B. The Y618A corresponds to a tyrosine to alanine change in position 618 in SCNN1B. All constructs were verified by sequencing.

### Cell culture and transfection

HEK 293T cells (ATCC CRL-3216, Manassas, VA) were cultured in growth media consisting of DMEM, 10% fetal bovine serum, 1% penicillin, and streptomycin. Cells were maintained in matrigel coated 75 cm^2^ cell culture flasks with 5% CO_2_ in a humidified 37°C incubator. For transfection with ENaC constructs, 1 × 10^6^ cells were seeded into a matrigel coated T25 flask. The following day, growth medium was replaced with 7 ml optiMEM. Then, a mix of 5 μl Lipofectamine 2000 with 250 μl optiMEM and separate mix of 1.5 μg plasmid with 250 μl optiMEM, were prepared. After incubating the mixtures separately for 5 min at room temperature they were combined, incubated for 25 min at room temperature then added to the cells. After 10 h of incubation at 37°C, the transfection media was replaced with growth media. After an additional 12–14 h incubation, the transfected cells were dissociated with Trypsin and 5 × 10^5^ cells were plated on a 35 mm matrigel coated glass bottom dish in growth medium. Cells were incubated at 37°C for an additional 24 h before imaging. For αβγ, SCNN1A, SCNN1B and SCNN1G were cotransfected in HEK 293T cells. Similarly, in the CFTR-ENaC interaction study, SCNN1A, SCNN1B SCNN1G and CFTR were cotransfected in HEK 293T cells. All experiments were performed at 37°C using a stage-top imaging incubator to avoid any temperature induced changes in endocytosis rates.

### Total internal reflection fluorescence microscopy

Objective-style TIRFM was utilized for all imaging studies. This setup is capable of detecting fluorophores on the plasma membrane and minimizes the fluorescence background from intracellular components. Dendra2 was excited with a 488 nm or 561 nm DPSS laser through a high numerical aperture objective (Olympus 1.49 NA 60× oil immersion, Tokyo, Japan). In order to obtain total internal reflection, the laser was focused on the back aperture of the objective lens and the angle was adjusted using a stepper motor to translate the beam laterally across the objective lens. An electron multiplying charge coupled device (EMCCD) (Andor iXon Ultra 897, Belfast, United Kingdom) was employed to detect the Dendra2 fluorescence signal.

### ENaC half-life measurement

Photoconvertable fluorescent protein (Dendra2) was utilized to measure the ENaC half-life on the plasma membrane. Before imaging, growth media in the glass bottom dish was replaced with Leibovitz’s L-15 with 100 μM ascorbic acid. At the start of each imaging session a control experiment was performed to correct for photobleaching during data collection. To obtain the correction curve, a randomly selected cell was photoconverted in TIRF with a 405 nm laser; One to two minute(s) after photoconvertion, 22 consecutive TIRF images were taken with 561 nm excitation (the total imaging time is less than 60 s). This was used to simulate the same level of laser exposure that occurs during the time-lapse imaging session. The fluorescence decay observed in these images results from 561 nm induced photobleaching. We assume on this time scale (< 60 s) that protein endocytosis is negligible. Several cells in the same dish were then identified and the *xy* location was recorded. TIRF images of these selected cells were taken before Dendra2 photoconvertion using both 561 nm and 488 nm excitation. This verified the absence of any fluorescence in the red emission channel prior to photoconversion. These cells were then exposed to TIRF-oriented 405 nm laser (~16.5 mW at the objective) excitation for 3 s. Medium in the glass bottom dish was changed from Leibovitz’s L-15 with 100 μM ascorbic acid to regular Leibovitz’s L-15 after photoconvertion taking care to not move the position of the dish. The presence of ascorbic acid during photoconversion and replacement with fresh media directly after photoconversion are important to maintain cell viability during time-lapse imaging. Real-time TIRF images were then acquired for both 561 nm and 488 nm excitation to collect emission in both the red and green channels at 20 min interval for a total of 7 h. Image collection was initiated 20 min after photoconversion to allow for the clearance of endosomes. For all time series, time 0 is the start of data collection taken 20 min after photoconversion. The intensity of both 488 nm and 561 nm lasers on the objective were ~1.0 mW and the 405 nm laser intensity on the objective was ~16.5 mW. The cells were kept at 37°C for the duration of the experiment using a stage top incubator. For control studies with dynasore, 80 μM dynasore was added to the imaging media containing ascorbic acid 20 min before imaging and the medium was then changed to Leibovitz’s L-15 with 80 μM dynasore after photoconvertion.

### Data analysis

Quantification of fluorescence integrated density (ID) was determined using ImageJ (NIH) by manually selecting an intensity-based threshold and region of interest. The decay in the fluorescence ID of each image series using the decay constructed from the control images where the decay resulted from photobleaching. This removed the decay related to photobleaching in each data set. The resulting fluorescence decay in each data set after correction was then attributed to the departure of the fluorescently labeled protein from the TIRF field of view due to endocytosis. The measured protein intensity data was initially fit to the exponential equation to determine the half-life (T_1/2_). All graphs show the mean with error bars representing standard error of the mean (SEM). *P* values were determined using a two-tailed *t* test with equal variance not assumed.

## RESULTS

**Figure 1.**
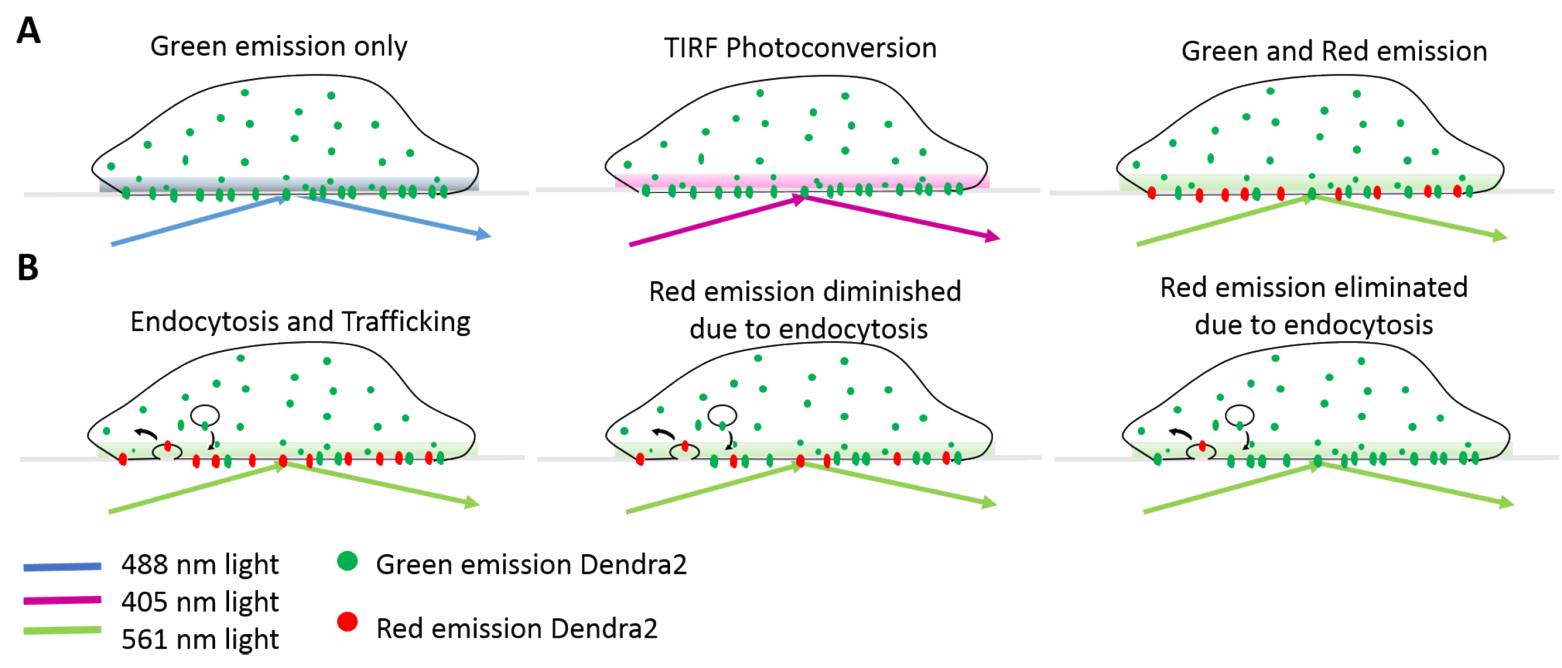
Outline of the method utilizing fluorescent protein, Dendra2, to measure PM protein half-life. **A.** The fluorescent behavior of cells expressing Dendra2 before and after photoconversion. **B.** The dynamic process of PM protein endocytosis and trafficking after portion of Dendra2 photoconverted to the red emissive form.

**Figure 2.**
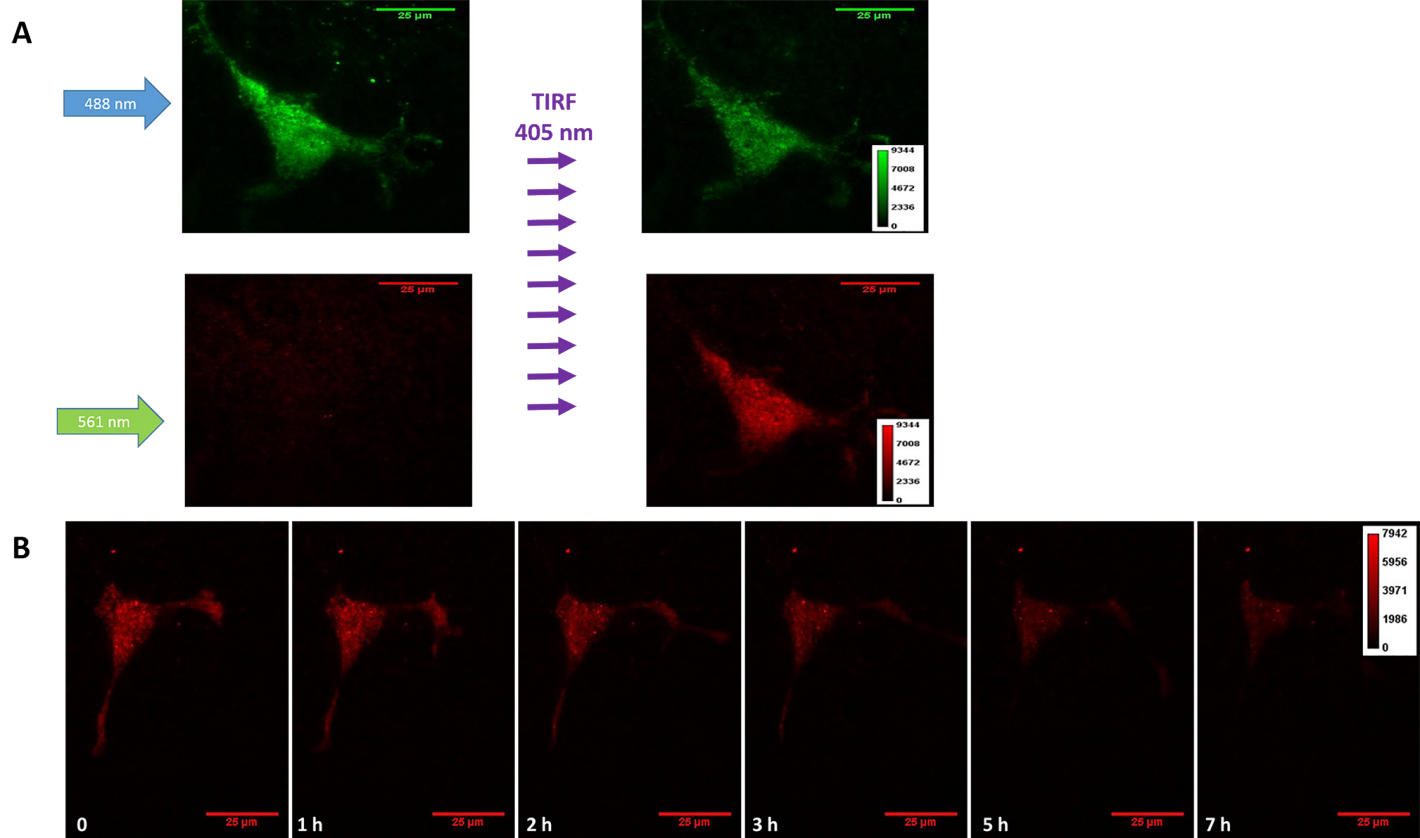
TIRF imaging of Dendra2. **A.** TIRF images of a single cell expressing α subunit of ENaC labelled with Dendra2 before and after photoconversion in both green and red emission channels with 488 nm and 561 nm excitation, respectively. **B.** Representative time trace TIRF images in the red emission channel at different time points. The color map in each row corresponds to all images in that row.

We developed a simple and straight-forward method to determine the half-life of membrane protein residence time on the plasma membrane with high temporal resolution. In response to exposure of the cells to 405 nm light, Dendra2 undergoes irreversible photoconversion where its green emission is switched to red [17]. Dendra2 is a long-lived protein and does not interfere with the degradation of the fused protein [18,19]. We expressed alpha-ENaC tagged with Dendra2 and selectively photoconverted the population residing at the plasma membrane with 405 nm TIRF excitation (**Fig. 1A**). This selectively generated a population of protein on the cell surface with red emission. Time-lapse imaging was used to monitor endocytosis of the membrane protein (**Fig. 1B**). The half-life of the protein on the plasma membrane was calculated by quantifying the decay in the red fluorescence over time. While new membrane protein is continuously delivered to the cell surface, it only exhibits green fluorescence and is not detected in the red channel. As seen in **Figure 2A**, cells are solely fluorescent in the green channel before exposure to 405 nm TIRF excitation. After exposure, fluorescence was observed in both the green and red channels, with the red fluorescence localized to the plasma membrane (**Fig. 2A**). The green fluorescence intensity was reduced after 405 nm exposure due to the conversion of a fraction of the population from green to red fluorophores. **Figure 2B** shows representative time-lapse TIRF images of the red emission. As the membrane receptor is internalized, the fluorescence diminishes due to departure from the TIRF observation volume.

**Figure 3.**
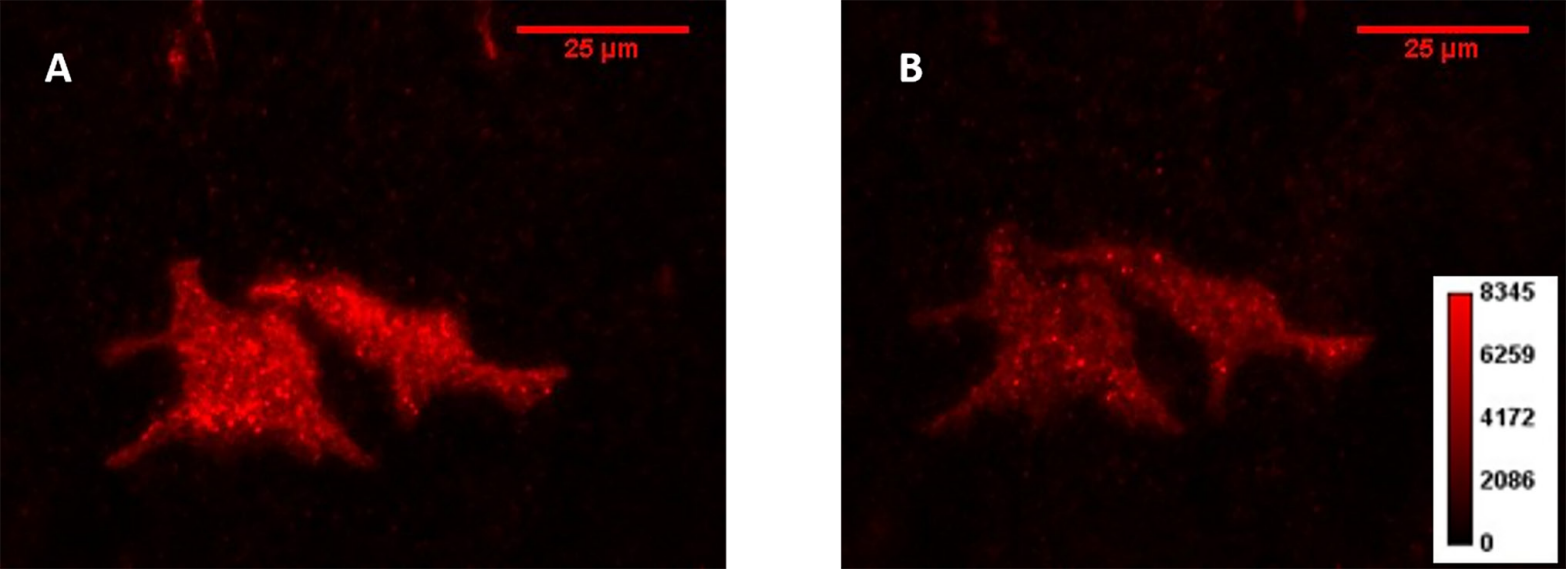
The fluorescence intensity comparison directly after conversion. **A.** Representative TIRF image of the red channel of Dendra2 labelled α, β and γ ENaC subunits showing the fluorescence intensity directly after photoconversion including the presence of endosomes. **B.** A TIRF image taken 20 min later showing the reduction in fluorescence intensity and clearance of endosomes. The color map corresponds to both images.

The TIRF field of view extends approximately 100 to 150 nm beyond the glass coverslip and is wavelength dependent. The 405 nm conversion light extends to a much shallower depth than either the 488 nm or 561 nm excitation light [20]. The plasma membrane is approximately 10 nm thick which means that the TIRF excitation field extends beyond the membrane and encompasses an area including endosomes that are budding or have recently budded off the cell surface. Studies have shown that fluorescently labeled clathrin-coated pits separating from the plasma membrane and imaged using TIRF microscopy remain in the excitation volume on average for less than a minute [21-23]. Endosomes remain coated with clathrin until separated from the plasma membrane by several hundred nanometers, well outside of the TIRF excitation volume [22]. This is supported by TIRF images taken directly after photoconversion where we observed small puncta indicating the presence of endosomes containing receptors (**Fig. 3A**). These punctate regions had cleared from the TIRF field of view 20 min after photoconversion (**Fig. 3B**). In order to account for the possibility that we are converting membrane receptors in endosomes along with those present on the plasma membrane, the first image used for our fluorescence decay time series is collected 20 min after activation. This first data collection point, 20 min post activation, is considered time 0 for all fluorescence decay measurements. This gives sufficient time for the clearance of all endosomes in the excitation volume. This time window allows us to selectively track changes in membrane protein residence on the plasma membrane. The half-life of each protein was quantified by fitting the fluorescence intensity over time to an exponential. Initiating fluorescence intensity measurements after a 20 min interval provides sufficient time for endosome clearance and does not affect the calculated half-life for measurements in the time frame of the proteins measured here.

The quantified fluorescence intensity versus time plots were fit with an exponential equation to calculate the half-life of the protein. We used this approach to measure the half-life of ENaC on the plasma membrane where time-lapse TIRF images were taken every 20 min. To verify that the loss of fluorescence intensity was due to endocytosis of the membrane receptor rather than lateral diffusion along the cell surface, we performed control experiments with dynasore, a small molecule dynamin inhibitor of GTPase activity which results in the inhibition of clathrin-mediated endocytosis in eukaryotic cells. During dynasore treatment endocytosis cannot occur and any observed decay in the red fluorescence results from other processes such as diffusion of the protein along the cell surface outside of the TIRF field of view. As shown in **Figure 5A**, α ENaC showed a slight decay (less than 15%) in the presence of dynasore indicating minimal loss of fluorescence due to diffusion or other sources. This indicates that the substantial fluorescence decay observed in the absence of dynasore can be attributed to endocytosis. Based on these studies, we assume that lateral diffusion has a negligible effect on the observed decrease in red fluorescence intensity.

**Figure 4.**
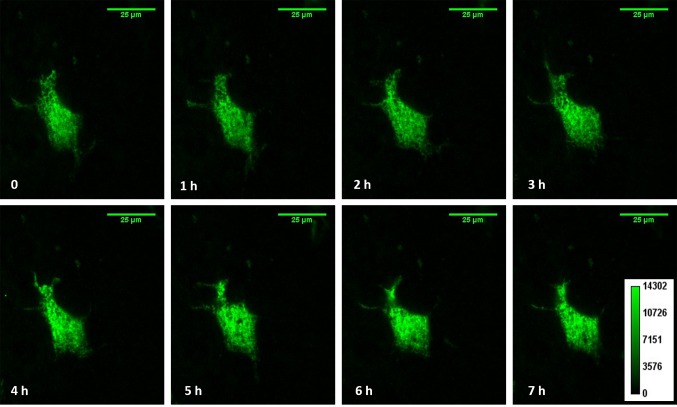
Fluorescence intensity of unconverted Dendra2 over time. Representative time trace TIRF images of cells expressing Dendra2 labelled α, β and γ subunits in the green emission channel at different time points. The color map corresponds to all images in this figure.

**Figure 5.**
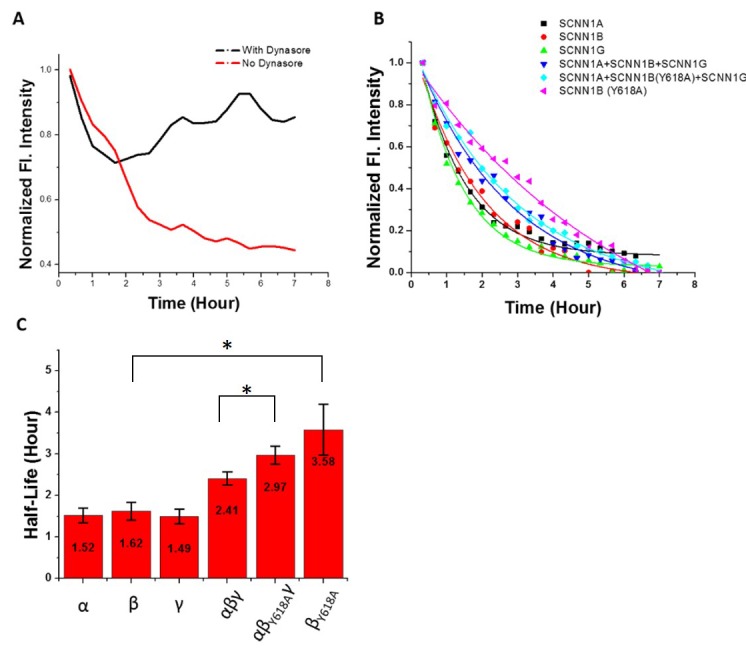
Half-life of ENaC subunits on the plasma membrane. **A.** Time course of red fluorescence intensity values of cells transfected with α ENaC -Dendra2 with (black) or without (red) dynasore treatment. The values at time 0 were normalized. **B.** Time course of red fluorescence intensity values of cells transfected with Dendra2 labelled different ENaC subunit(s). Values were fitted to a single exponential equation and the values at time 0 were normalized. Time 0 starts at the first image 20 min after photoconversion. **C.** Bar plot of ENaC subunits half-life. Values are average ± SE (10–15 cells were measured for each experiment). This shows an increase in the turnover of the β only receptor (column 2) and the mutant β subunit (column 6) and between the heteromer containing all subunits with a non-mutated β subunit (column 4) versus that with a mutation in the β subunit (column 5). *T*-tests (*P* = 0.05) were performed with unequal variance assumed (*P < 0.05).

We measured the plasma membrane half-life of α, β, and γ subunits on their own. The subunits exhibited similar residence time on the cell surface where α, β, and γ gave a half-life of 1.52 h, 1.62 h and 1.49 h, respectively (**Fig. 5C**). However, when α, β, and γ were expressed together, we observed a significantly longer half-life (2.41 h) than any of the subunits alone. The subunits are thought to form active ENaC channels with a 1:1:1 stoichiometry. A mutation in the β subunit of ENaC has been associated with Liddle’s syndrome which is a hereditary disorder that results in severe hypertension [24]. The mutation, β_Y618A_, occurs in the PY motif (PPxY) of the β subunit, which serves as the binding site for a protein ubiquitin E3 ligase such as nedd4. Interaction with the E3 ligase is responsible for reducing cell surface population of ENaC [12,25]. The mutation disrupts interactions between the ligase and the subunit leading to a longer half-life on the cell surface [13,26]. We generated β ENaC containing the Y618A mutations and compared the half-life versus wild-type protein. The mutation increased the half-life to 3.58 h, more than twice that for the wild type subunit. We also compared the half-life when α and γ were expressed with the mutant β. While we saw a statistically significant increase in the residence time on the cell surface, the presence of the other subunits compensated for the effect of the mutation. The half-life of αβ_Y618A_γ was 2.97 h, while the half-life of αβγ was 2.41 h. **Figure 5B** plots the red fluorescence intensity values over time starting 20 min after photoconversion. The half-lives calculated in **Figure 5C** were based on the red fluorescence decay curves of **Figure 5B**. We also monitored the fluorescence in the green emission channel. As seen in **Figure 4**, the green fluorescence intensity remained stable and in focus for the duration of the time series. Thus, the decay in the red fluorescence is not due to the loss of optical focus or change in cell morphology.

**Figure 6.**
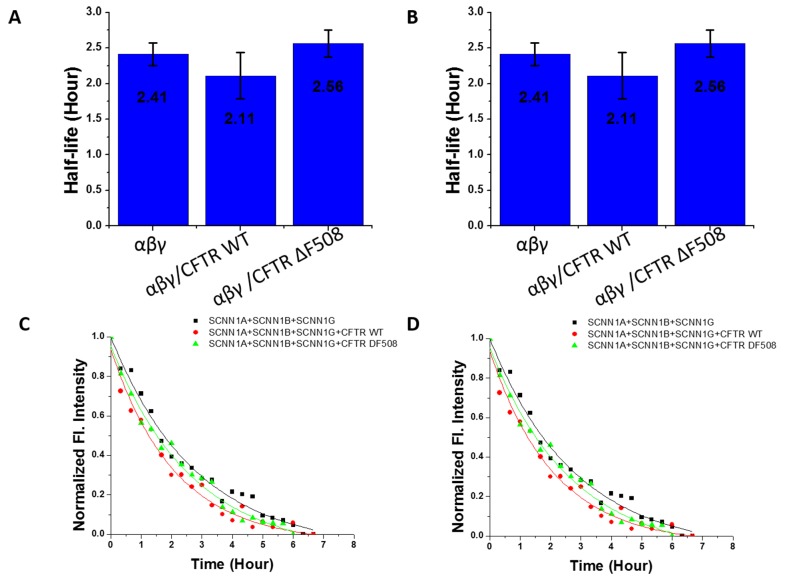
The influence of wild type and ΔF508 CFTR on ENaC half-life. **A** and **B.** Bar diagrams of half-life of ENaC subunit(s) with or without CFTR. Values are average ± SE (10–15 cells were measured for each experiment). Two tails *t*-tests (*P* = 0.05) were performed with unequal variance assumed. **C** and **D.** Time course of red fluorescence intensity values of the cells expressing Dendra2 labelled ENaC subunit(s) with or without CFTR. Values were fitted to a single exponential equation and the values at time 0 were normalized.

Previous studies have shown that the cystic fibrosis transmembrane regulator (CFTR) interacts through scaffold proteins to indirectly regulate ENaC. Loss of CFTR increases ENaC channel activity and possibly alters protein trafficking [27,28]. To determine if this interaction affected ENaC residence time on the cell surface, we measured the half-life of ENaC in the presence of wild-type CFTR and in the presence of a CFTR mutation (ΔF508) that prevents it from reaching the cell surface. CFTR wild-type or ΔF508 were cotransfected with ENaC in HEK 293T cells. As seen in **Figure 6A** and **6B**, we measured the turnover of α ENaC in the presence of CFTR WT and CFTR ΔF508 and found half-lives of 1.49 h and 1.54 h, respectively. These results were not significantly different from that of α ENaC alone (1.52 h). We also measured the half-life of αβγ with CFTR wild type (2.11 h) and CFTR ΔF508 (2.56 h). These results were also not significantly different from that of αβγ (2.41 h). This indicates that the bridging scaffold proteins between CFTR and ENaC do not sense the loss of CFTR by influencing the residence time of ENaC on the cell surface. **Figure 6C** and **6D** show the time course of red fluorescence intensity values of the cells expressing Dendra2 labelled ENaC subunit(s) with or without CFTR.

## DISCUSSION

We present a new approach to quantify the half-life of membrane proteins on the cell surface, through labelled them with the photoconvertible fluorescent protein, Dendra2. Previous studies have utilized photoconvertible fluorescent proteins to monitor transport between organelles [29], protein degradation [30], and protein translation [19]. In order to limit excitation and photoconversion to only the plasma membrane, we utilized TIRF microscopy. This enabled us to extend the application of Dendra2 to study the half-life of membrane proteins on the plasma membrane.

Using this approach, we measured the half-life of different ENaC subunits and mutations. ENaC has been reported to turn over rapidly on the cell surface, with a reported half-life ranging from 40–120 min in cultured cells [12,26]. The apparent lifetime on the membrane was highly dependent on the technique used for the measurement. Yu *et al*. performed single-channel measurements to quantify the half-life of ENaC and reported that the average half-life in CHX-treated cells was 1.45 ± 0.24 h, while it was 3.28 ± 0.89 h with puromycine treatment [9].

Using the Dendra2 based technique, we measured the lifetime for ENaC α, β and γ subunits at 1.52 h, 1.62 h, and 1.49 h, respectively indicating near identical stability on the plasma membrane for each subunit in the absence of the others. However, when all 3 subunits are co-expressed this seemed to increase the stability of ENaC on the cell surface. We observed a shift in the lifetime from approximately 1.5 h to 2.4 h. This change likely results from ENaC assembling as a heterotrimer rather than a homo trimer. It has also been reported that a mutation in the PY motif in the β subunit can cause a longer half-life on the plasma membrane [31]. Our measurements were consistent with this. We observed a 3.58 h half-life of β_Y618A_ alone which is more than a two-fold increase in the half-life over β wild type. Interestingly, we observed only a moderate increase (2.4 to 3 h) in the half-life on the plasma membrane when wild type α and γ were coexpressed with β_Y618A_ as compared to the same combinations with wild type β. In the absence of the other subunits it appears that this mutation is sufficient to disrupt interactions with cellular machinery responsible for the endoctytosis of ENaC. In a heterotrimer, the presence of the other subunits moderates the effect of the mutation.

The results described here illustrate that the temporal resolution of this technique can be used to quantitatively monitor changes in membrane receptor half-life on the plasma membrane. We have applied the same technique to membrane receptors with a longer half-life. For example, we observed a decay rate for α4β2 nicotinic receptors that matches well with previous studies using other techniques (data not shown). The Dendra2 based approach was simple and straight forward as compared to currently used techniques such as biotinylation western blotting, radioactive pulse-chase and electrophysiology approach which have more complicated experimental procedures. The method described here is much less labor intensive and performs with a higher temporal resolution compared to existing techniques. Additionally, we were able to avoid potential errors caused by compounds that alter cellular metabolism which are required for many of the existing techniques.
